# Effect of Oral Taurine on Morbidity and Mortality in Elderly Hip Fracture Patients: A Randomized Trial

**DOI:** 10.3390/ijms160612288

**Published:** 2015-05-29

**Authors:** Mireille F. M. Van Stijn, Arnoud A. Bruins, Mechteld A. R. Vermeulen, Joost Witlox, Tom Teerlink, Margreet G. Schoorl, Jean Pascal De Bandt, Jos W. R. Twisk, Paul A. M. Van Leeuwen, Alexander P. J. Houdijk

**Affiliations:** 1 Department of Surgery, Medical Center Alkmaar, Wilhelminalaan 12, 1815 JD Alkmaar, The Netherlands; E-Mails: m.f.vanstijn@amc.uva.nl (M.F.M.V.S.); arnoudbruins@hotmail.com (A.A.B.); 2 Academic Medical Center Amsterdam, Department of Anesthesiology, Meibergdreef 9, 1105 AZ Amsterdam, The Netherlands; 3 VU University Medical Center Amsterdam, Department of Surgery, De Boelelaan 1117, 1081 HV Amsterdam, The Netherlands; E-Mails: mar.vermeulen@vumc.nl (M.A.R.V.); pam.vleeuwen@vumc.nl (P.A.M.V.L.); 4 VU University Medical Center Amsterdam, Internal Medicine, De Boelelaan 1117, 1081 HV Amsterdam, The Netherlands; 5 Medical Center Alkmaar, Geriatric Medicine, Wilhelminalaan 12, 1815 JD Alkmaar, The Netherlands; E-Mail: j.witlox@mca.nl; 6 VU University Medical Center Amsterdam, Clinical Chemistry, De Boelelaan 1117, 1081 HV Amsterdam, The Netherlands; E-Mail: t.teerlink@vumc.nl; 7 Medical Center Alkmaar, Clinical Chemistry, Hematology & Immunology, Wilhelminalaan 12, 1815 JD Alkmaar, The Netherlands; E-Mail: m.g.schoorl@mca.nl; 8 Université Paris Descartes, Sorbonne Paris Cité, EA4466 Paris, France; E-Mail: jean-pascal.de-bandt@univ-paris.fr; 9 VU University Medical Center Amsterdam, Epidemiology and Biostatistics, De Boelelaan 1117, 1081 HV Amsterdam, The Netherlands; E-Mail: jwr.twisk@vumc.nl; 10 Trial Center Holland Health, Kennemerstraatweg 10, 1817 MS Alkmaar, The Netherlands

**Keywords:** oxidative stress, taurine supplementation, surgery, elderly patients

## Abstract

Hip fracture patients represent a large part of the elderly surgical population and face severe postoperative morbidity and excessive mortality compared to adult surgical hip fracture patients. Low antioxidant status and taurine deficiency is common in the elderly, and may negatively affect postoperative outcome. We hypothesized that taurine, an antioxidant, could improve clinical outcome in the elderly hip fracture patient. A double blind randomized, placebo controlled, clinical trial was conducted on elderly hip fracture patients. Supplementation started after admission and before surgery up to the sixth postoperative day. Markers of oxidative status were measured during hospitalization, and postoperative outcome was monitored for one year after surgery. Taurine supplementation did not improve in-hospital morbidity, medical comorbidities during the first year, or mortality during the first year. Taurine supplementation lowered postoperative oxidative stress, as shown by lower urinary 8-hydroxy-2-deoxyguanosine levels (Generalized estimating equations (GEE) analysis average difference over time; regression coefficient (Beta): −0.54; 95% CI: −1.08–−0.01; *p* = 0.04), blunted plasma malondialdehyde response (Beta: 1.58; 95% CI: 0.00–3.15; *p* = 0.05) and a trend towards lower lactate to pyruvate ratio (Beta: −1.10; 95% CI: −2.33–0.12; *p* = 0.08). We concluded that peri-operative taurine supplementation attenuated postoperative oxidative stress in elderly hip fracture patients, but did not improve postoperative morbidity and mortality.

## 1. Introduction

The increase in the number of elderly hip fracture patients is a growing medical concern. Severe morbidity and age-controlled excessive mortality rates are reported when these patients undergo surgery. In-hospital mortality rates of 5.4% rise to 20% at six months, and 30% at one year [1]. Mortality further increases in case of complications, e.g., by a wound infection during the postoperative period [2].

Studies on the effect of nutrition in elderly hip fracture patients have shown promising results in terms of clinical outcome. An increased attention to dietary intake and nutritional status in elderly women with a hip fracture reduced mortality during hospital stay and after four months follow up, by 60% and 43% respectively [3]. In addition, intravenous and oral nutritional supplementation with a mixture of carbohydrate, protein, vitamins and minerals, lowered postoperative infection rates in hip fracture patients [4]. Unfortunately, because of the use of a combination of nutrients and the lack of adequate control groups, these studies do not reveal an underlying target mechanism.

Oxidative stress, induced by surgical tissue injury, is seen as part of the surgical stress response that is associated with postoperative morbidity and mortality [5]. Especially elderly patients are prone to oxidative stress, because their insufficient dietary habits lead to low antioxidant status [6,7]. Hence, oxidative stress might be a target for intervention in elderly surgical patients.

Taurine, a semi-essential amino acid with antioxidant action, reduces oxidative stress in animals and humans [8,9]. In hyperglycemic rats taurine supplementation reduced markers of lipid peroxidation and prevented the associated insulin resistance [8]. During coronary artery bypass surgery in humans, the intravenous administration of taurine reduced lipid peroxidation, decreased cell damage and improved mitochondrial survival at the time of reperfusion [9]. Compared to the young, taurine plasma levels are 43% lower in the geriatric population [10]. Taurine may therefore be useful in reducing postoperative oxidative stress.

In trauma patients, enteral nutritional supplementation with glutamine, the precursor of glutathione, lowered infectious morbidity possibly by affecting the immuno-inflammatory response [11,12]. Interestingly, glutamine enrichment in these trauma patients increased plasma taurine levels [13], which may have contributed to the positive effects by reducing oxidative stress.

The aim of this study was to evaluate whether taurine supplementation may improve clinical outcome in the elderly hip fracture patient by reducing oxidative stress.

## 2. Results and Discussion

### 2.1. Results

#### 2.1.1. Patients

**Figure 1:**
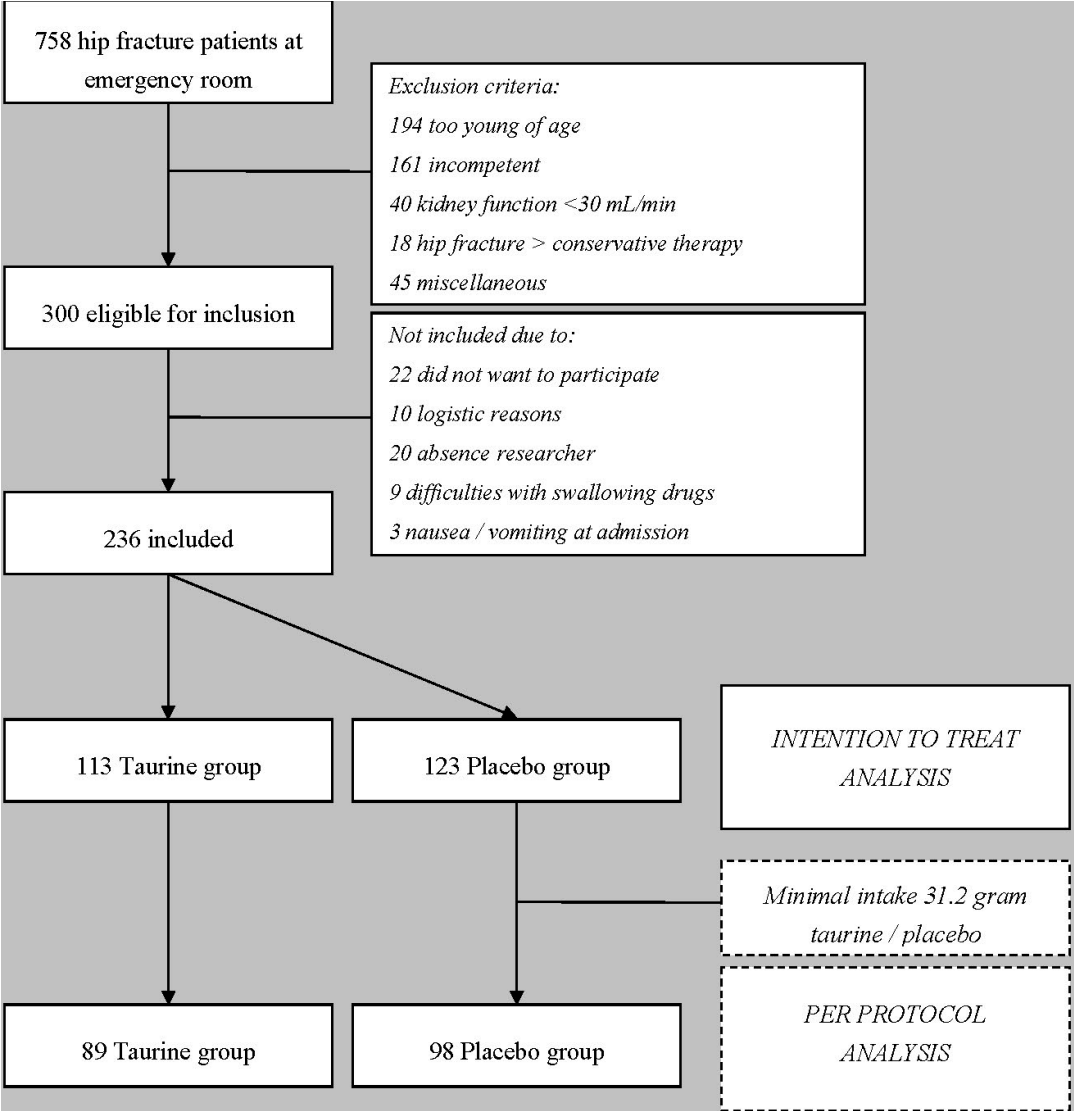
Flow Chart Inclusion. Kidney function is defined as creatinine clearance calculated with the Cockroft-Gault formula; miscellaneous patients: Three gastro-intestinal passage disorder, three language barrier, one hearing disability, thirteen secondary fractures, two had surgery in other hospital, eight cancer ± metastases, five primary total hip arthroplasty, six already participating in current study trial with contra lateral hip, three planned transfer to other hospital directly after surgery, one participating in other study trial.

Between March 2008 and July 2010, 236 primary hip fracture patients aged 75 years or older entered the study. The flow chart of inclusion in the study is shown in Figure 1. Baseline patient characteristics of the taurine and placebo group are shown in Table 1, on an intention to treat analysis. Per protocol analysis of the baseline characteristics revealed no other differences (data not shown).

Taurine and placebo capsules were well tolerated and no side effects were observed. The intake of taurine resulted in a higher plasma taurine concentration compared to the placebo group, which remained elevated during the postoperative period (GEE analysis average difference over time; regression coefficient (Beta): 701; 95% confidence interval (95% CI): 578–824; *p* = 0.00) (Table 2). The majority of patients in the taurine and placebo group were well nourished at admission according to the Mini Nutritional Assessment and there were no differences between the groups (Table 1). In the postoperative period dietary intakes as breakfast, lunch and dinner were scored using the “Nutrition Day in Europe form 3b” and data are shown in Table 3. No differences between the groups were observed at breakfast, lunch and dinner when addressing the average difference over time. For breakfast, lunch and dinner the majority of patients ate 50% or more of their meals.

**Table 1:** Baseline characteristics.

Parameter	Taurine	Placebo	*p*-Value
**Male/Female**	33/80	30/93	0.41
**Age (years)**	84.4 ± 5.4	84.4 ± 4.9	0.95
**BMI (kg/m^2^)**	24.1 ± 4.0	24.5 ± 3.8	0.35
**Barthel ADL index (max. 20 points)**	17 ± 3	18 ± 3	0.20
**Hemoglobin (mmol/L)**	7.7 ± 0.9	7.8 ± 0.9	0.43
Creatinine clearance * (mL/min)	56 ± 19	55 ± 23	0.90
**Vitamin B6 (nmol/L)**	83.7 ± 73.4	80.1 ± 51.6	0.67
**Albumin (g/L)**	34.5 ± 4.7	35.1 ± 3.6	0.35
**Mini Nutritional Assessment**			
Screening score (max. 14 points)	11.6 ± 2.7	11.9 ± 2.8	0.39
Assessment (max. 16 points)	12.5 ± 1.7	12.7 ± 1.9	0.52
Total Assessment (max. 30 points)	24.2 ± 3.8	24.6 ± 4.3	0.39
Nutritional Status **			0.40
Malnourished, *n*/*N* (%)	6/109 (5)	7/122 (6)	
At risk of malnutrition, *n*/*N* (%)	29/109 (27)	24/122 (20)	
Well nourished, *n*/*N* (%)	74/109 (68)	91/122 (74)	
**Medical comorbidities (quantity)**	2.1 ± 1.6	2.1 ± 1.3	0.93
0, *n*/*N* (%)	14/113 (12)	12/123 (10)	
1*n*/*N* (%)	33/113 (29)	33/123 (27)	
2, *n*/*N* (%)	29/113 (26)	32/123 (26)	
≥3, *n*/*N* (%)	37/113 (33)	46/123 (37)	
Cardiovascular disease, *n*/*N* (%)	108/113 (95)	113/123 (92)	0.76
Cerebral vascular disease, *n*/*N* (%)	20/113 (18)	17/123 (14)	0.41
Respiratory disease, *n*/*N* (%)	11/113 (10)	11/123 (9)	0.83
Diabetes, *n*/*N* (%)	24/113 (21)	24/123 (19)	0.74
Musculoskeletal, *n*/*N* (%)	34/113 (30)	27/123 (22)	0.24
Other, *n*/*N* (%)	40/113 (35)	64/123 (52)	0.04
**Concomitant Medication (quantity)**	5 ± 4	5 ± 4	0.61
0–1, *n*/*N* (%)	23/113 (20)	24/123 (20)	
2–5, *n*/*N* (%)	45/113 (40)	60/123 (49)	
6–10, *n*/*N* (%)	37/113 (33)	30/123 (24)	
>10, *n*/*N* (%)	8/113 (7)	9/123 (7)	
**ASA Score**			
1, *n*/*N* (%)	19/113 (17)	22/123 (18)	0.17
2, *n*/*N* (%)	50/113 (44)	67/123 (54)	
3, *n*/*N* (%)	44/113 (39)	34/123 (28)	
**Type of Surgery**			
Hemiarthroplasty, *n*/*N* (%)	48/110 (44)	65/122 (53)	0.08
Cannulated screws, *n*/*N* (%)	4/110 (4)	7/122 (6)	
Sliding hip screw, *n*/*N* (%)	7/110 (6)	7/122 (6)	
Gamma nail, *n*/*N* (%)	51/110 (46)	43/122 (35)	
**Type of Anesthesia**			
Spinal, *n*/*N* (%)	89/110 (81)	93/122 (76)	0.33
Spinal + general, *n*/*N* (%)	2/110 (2)	1/122 (1)	
General, *n*/*N* (%)	19/110 (17)	28/122 (23)	
**Time of Admission to Surgery (h)**	20.2 ± 15.0	21.4 ± 20.6	0.61
**Duration of surgery (min)**	49 ± 25	48 ± 18	0.69

Intention to treat analysis; data are presented by mean ± SD unless otherwise stated; *n*/*N* = number with characteristic/total number; % = percentage; BMI indicates body mass index; Barthel ADL index, Barthel activities of daily living index; Hb, hemoglobin; * creatinine clearance was calculated using the Cockroft-Gault formula; ** Nutritional status according to Mini Nutritional Assessment cut-off points (malnourished: <17 points, at risk of malnutrition: 17 to 23.5 points, well nourished: ≥24 points); ASA score, American Society of Anesthesiologists score.

**Table 2:** Surgical stress response in a sub group of 60 patients.

Group		Preoperative	Postoperative		*Beta* (95% CI) p *
			Day 1	Day 5	
**WBC (10^9^/L)**	**T**	8.0 (3.4–16.9)	9.8 (4.5–16.1)	8.1 (2.7–11.4)	−0.13 (−1.07–0.81) 0.78
	**P**	9.2 (4.4–18.2)	9.9 (5.2–15.9)	7.4 (3.5–13.7)	
**CRP (mg/L)**	**T**	4 (1–25)	85 (19–229)	66 (22–218)	−1.2 (−27.0–24.5) 0.93
	**P**	4 (1–117)	103 (8–209)	83 (5–207)	
**IL-6 (pg/mL)**	**T**	29.1 (5.5–415.0)	104.5 (11.9–335.3)	21.7 (6.7–360.0)	6.8 (−17.8–31.4) 0.59
	**P**	39.6 (4.5–116.7)	91.4 (31.5–622.7)	18.1 (3.8–57.7)	
**Taurine (µmol/L)**	**T**	102 (49–474)	689 (169–1629)	685 (97–1561)	701 (578–824) 0.00
	**P**	96 (64–142)	86 (57–152)	74 (53–137)	
**Vitamin C (µmol/L)**	**T**	56 (27–135)	53 (15–104)	56 (18–92)	2.2 (−5.5–10.1) 0.57
	**P**	54 (26–107)	52 (26–79)	55 (26–97)	
**Vitamin E (µmol/L)**	**T**	35 (15–52)	26 (12–40)	28 (22–40)	−1.3 (−3.9–1.2) 0.31
	**P**	34 (18–56)	27 (16–48)	30 (17–45)	
**β-carotene (µmol/L)**	**T**	0.29 (0.08–1.07)	0.24 (0.07–0.70)	0.22 (0.08–0.81)	−0.02 (−0.06–0.02) 0.27
	**P**	0.28 (0.12–0.89)	0.23 (0.07–0.71)	0.27 (0.11–1.04)	
**Glutathione (µmol/L)**	**T**	767 (256–1350)	683 (223–1195)	651 (161–1035)	2.5 (−59.8–64.9) 0.94
	**P**	745 (505–1228)	696 (455–1006)	637 (336–998)	
**Ox. LDL/apoB100 ratio (U/g)**	**T**	94 (76–130)	100 (81–145)	117 (96–146)	2.39 (−2.39–7.17) 0.33
	**P**	100 (69–126)	106 (80–139)	117 (84–150)	
**Malondialdehyde (µmol/L) ^‡^**	**T**	7.0 (4.3–10.8)	9.3 (6.1–19.1)	7.6 (5.4–12.7)	−0.45 (−1.64–0.74) 0.46
	**P**	7.3 (4.6–24.0)	10.8 (6.4–23.3)	7.5 (4.8–11.6)	
**F2-Isoprostane (pmol/mmol creatinine)**	**T**	106.2 (23.8–359.3)	76.7 (11.5–184.6)	115.9 (47.2–184.2)	−1.62 (−18.5–15.2) 0.85
	**P**	113.7 (25.1–434.1)	82.0 (21.9–233.9)	109.0 (46.7–275.9)	
**8OHdG (nmol/mmol creatinine)**	**T**	2.4 (1.0–7.4)	1.9 (0.7–6.8)	2.5 (0.3–5.5)	−0.54 (−1.07–−0.01) 0.04
	**P**	2.0 (0.8–10.4)	2.3 (0.8–12.8)	2.2 (1.1–8.8)	
**Lactate (µmol/L)**	**T**	833 (323–1540)	1211 (744–4797)	1257 (632–3347)	−44 (−347–258) 0.77
	**P**	912 (467–2861)	1568 (738–6787)	1159(647–2092)	
**Pyruvate (µmol/L)**	**T**	48 (25–113)	81 (50–242)	86 (49–164)	0.63 (−16.7–17.9) 0.94
	**P**	52 (20–200)	86 (49–275)	75 (35–138)	
**Lactate/Pyruvate Ratio**	**T**	18 (12–29)	16 (10–20)	14 (10–20)	−1.10 (−2.33–0.12) 0.08
	**P**	18 (9–33)	14 (12–25)	16 (10–22)	

T = taurine; P = placebo; data are presented by median [range]; WBC = white blood cell count; CRP = C-reactive protein; IL-6 = interleukin 6; Ox. LDL = oxidized low density lipoprotein; apoB100 = apolipoprotein-B100; 8OHdG = 8-hydroxy-2-deoxyguanosine; Intention to treat analysis; GEE analysis was used for statistical analysis, where the Beta is the regression coefficient and indicates the difference between the groups. A positive Beta indicates that the average parameter level in the taurine group is higher than in the placebo group (e.g., for taurine levels), whereas a negative value indicate that the average parameter level is higher in the placebo group (e.g., for lactate), but whether this difference is significant is indicated by the *p*-value; * *p* = *p*-value of an average difference over time between taurine and placebo group, corrected for baseline; **^‡^** there is a significant difference between the groups in the change over time GEE analysis; (95% CI) means 95% confidence interval.

**Table 3:** Postoperative dietary intake according to “Nutrition Day in Europe”.

Group		Day 1 *n*/*N* (%)	Day 2 *n*/*N* (%)	Day 3 *n*/*N* (%)	Day 4 *n*/*N* (%)	Day 5 *n*/*N* (%)	* *p*-Value
**Nutrition Day Breakfast**							
All eaten	**T**	57/77 (74)	55/73 (75)	65/75 (87)	55/70 (79)	48/57 (84)	0.42
	**P**	62/93 (67)	73/93 (78)	80/93 (86)	61/77 (79)	56/64 (87)	
50% Eaten	**T**	12/77 (16)	9/73 (12)	7/75 (9)	10/70 (14)	5/57 (9)	
	**P**	7/93 (7)	10/93 (11)	8/93 (9)	10/77 (13)	5/64 (8)	
25% Eaten	**T**	6/77 (8)	5/73 (7)	2/75 (3)	4/70 (6)	4/57 (7)	
	**P**	14/93(15)	4/93 (4)	4/93 (4)	1/77 (1)	2/64 (3)	
Nothing	**T**	2/77 (2)	4/73 (6)	1/75 (1)	1/70 (1)	0/57 (0)	
	**P**	10/93 (11)	6/93 (7)	1/93 (1)	5/77 (7)	1/64 (2)	
**Nutrition Day Lunch**							
All eaten	**T**	48/73 (66)	57/72 (79)	52/68 (77)	48/67 (72)	36/44 (82)	0.83
	**P**	57/89 (64)	67/87 (77)	69/80 (86.5)	54/69 (78)	51/58 (88)	
50% Eaten	**T**	15/73 (21)	8/73 (11)	9/68 (13)	15/67 (22)	5/44 (11)	
	**P**	16/89 (18)	6/87 (7)	5/80 (6)	8/69 (12)	4/58 (7)	
25% Eaten	**T**	9/73 (12)	5/72 (7)	3/68 (4)	2/67 (3)	1/44 (2)	
	**P**	7/89 (8)	8/87 (9)	5/80 (6)	3/69 (4)	1/58 (2)	
Nothing	**T**	1/73 (1)	2/72 (3)	4/68 (6)	2/67 (3)	2/44 (5)	
	**P**	9/89 (10)	6/87 (7)	1/80 (1.5)	4/69 (6)	2/58 (3)	
**Nutrition Day Dinner**							
All eaten	**T**	30/65 (46)	33/62 (53)	34/64 (53)	29/58 (50)	26/40 (65)	0.38
	**P**	29/74 (39)	37/72 (51.5)	45/71 (63)	43/66 (65)	31/48 (65)	
50% eaten	**T**	17/65 (65)	16/62 (26)	16/64 (25)	21/58 (36)	5/40 (13)	
	**P**	27/74 (37)	21/72 (29)	19/71 (27)	15/66 (23)	12/48 (25)	
25% eaten	**T**	15/65 (23)	11/62 (18)	12/64 (19)	6/58 (10)	8/40 (20)	
	**P**	12/74 (16)	13/72 (18)	5/71 (7)	5/66 (8)	3/48 (6)	
Nothing	**T**	3/65 (5)	2/62 (3)	2/64 (3)	2/58 (4)	1/40 (2)	
	**P**	6/74 (8)	1/72 (1.5)	2/71 (3)	3/66 (4)	2/48 (4)	

T = taurine; P = placebo; intention to treat analysis; data are presented by *n*/*N* (%), whereby *n* = number with characteristic, *N* = total number, % = percentage; GEE analysis was used for statistical analysis; * *p*-value = *p*-value of an average difference over time between taurine and placebo group, corrected for baseline.

#### 2.1.2. Postoperative Morbidity and Mortality

**Figure 2:**
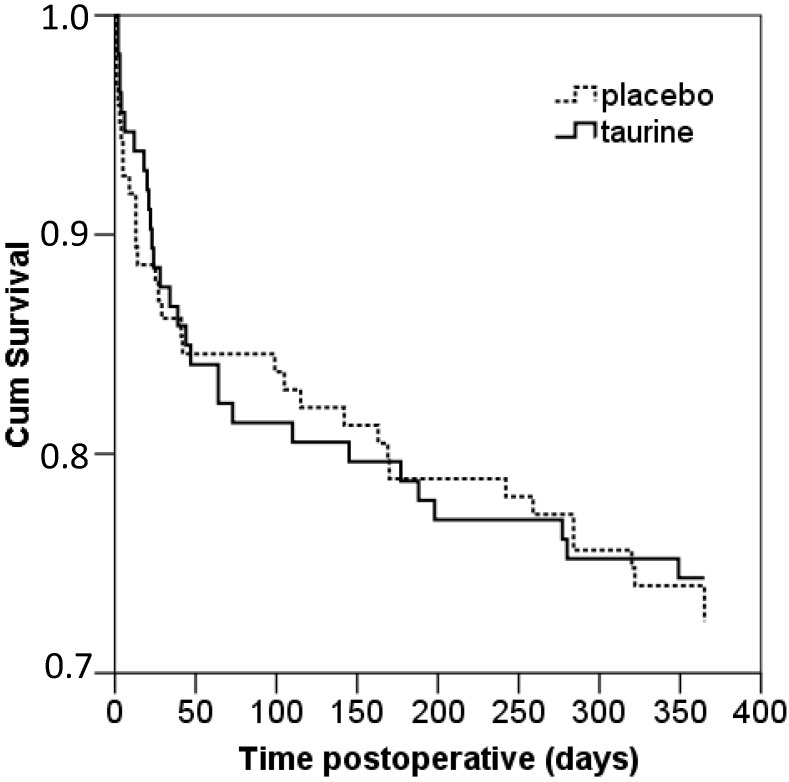
Kaplan Meier curve for mortality; 365 days after surgery. Intention to treat analysis; Log-rank = 0.75.

Data on postoperative morbidity and mortality are shown in Table 4 and Figure 2.

**Table 4:** Outcome parameters.

Parameter	Taurine	Placebo	*p*-Value
**Intake Supplement (grams)**	33.0 ± 9.7	33.3 ± 9.5	0.82
**Length of Hospital Stay (days)**	13 ± 10	13 ± 11	0.83
**In-Hospital Morbidity (quantity)**	1.0 ± 1.1	1.0 ± 1.1	0.95
0, *n*/*N* (%)	45/110 (41)	55/122 (45)	
1, *n*/*N* (%)	38/110 (34)	30/122 (25)	
2, *n*/*N* (%)	15/110 (14)	25/122 (20)	
≥3, *n*/*N* (%)	12/110 (11)	12/122 (10)	
Infectious, *n*/*N* (%)	11/110 (10)	18/122 (15)	0.54
Cardiovascular event, *n*/*N* (%)	5/110 (5)	13/122 (11)	0.12
Cerebral vascular accident, *n*/*N* (%)	1/110 (1)	2/122 (2)	0.62
Delirium, *n*/*N* (%)	26/110 (24)	27/122 (22)	0.79
Blood transfusion, *n*/*N* (%)	19/110 (17)	20/122 (16)	0.86
Reoperation or surgery otherwise, *n*/*N* (%)	6/110 (5)	6/122 (5)	0.91
Other, *n*/*N* (%)	40/110 (36)	35/122 (29)	0.33
**Barthel ADL index (max. 20 points)**			0.90 *
3 months follow-up	16 ± 4	17 ± 4	0.83 ^‡^
6 months follow-up	17 ± 4	17 ± 3	0.95 ^‡^
9 months follow-up	17 ± 3	17 ± 4	0.53 ^‡^
12 months follow-up	17 ± 4	17 ± 4	0.58 ^‡^
**Medical Comorbidities in First Year (quantity)**	0.8 ± 0.9	0.8 ± 0.8	0.82
0, *n*/*N* (%)	48/107 (44)	51/117 (44)	
1, *n*/*N* (%)	36/107 (34)	46/117 (39)	
2, *n*/*N* (%)	19/107 (18)	14/117 (12)	
≥3, *n*/*N* (%)	4/107 (4)	6/117 (5)	
Infectious, *n*/*N* (%)	30/107 (28)	29/117 (25)	0.69
Cardiovascular event, *n*/*N* (%)	7/107 (7)	8/117 (7)	0.93
Cerebral vascular accident, *n*/*N* (%)	0/107 (0)	2/117 (2)	0.17
Thromboembolic event, *n*/*N* (%)	1/107 (1)	2/117 (2)	0.61
Reoperation or surgery otherwise, *n*/*N* (%)	16/107 (15)	15/117 (13)	0.64
Anemia with medical intervention, *n*/*N* (%)	2/107 (2)	7/117 (6)	0.12
Cognitive impairment, *n*/*N* (%)	7/107 (7)	8/117 (7)	0.93
Other, *n*/*N* (%)	24/107 (22)	21/117 (18)	0.40
**Mortality Overall, *n*/*N* (%)**	23/107 (21)	27/116 (23)	0.75
In-hospital, *n*/*N* (%)	11/111 (10)	11/123 (9)	0.80
At 3 months follow-up, *n*/*N* (%)	16/108 (15)	16/119 (13)	0.77
At 6 months follow-up, *n*/*N* (%)	20/108 (18)	22/119 (18)	0.99
At 9 months follow-up, *n*/*N* (%)	22/108 (20)	23/119 (19)	0.84
At 12 months follow-up, *n*/*N* (%)	23/107 (21)	27/116 (23)	0.75

Intention to treat analysis; data are presented by mean ± SD unless otherwise stated; *n*/*N* = number with characteristic/total number, % = percentage; * *p*-Value of an average difference over time between taurine and placebo group, corrected for baseline using GEE analysis; ^**‡**^ *p*-value of a change over time between taurine and placebo group, corrected for baseline using GEE analysis.

In-hospital morbidity was not different between the groups with almost half the patients in both groups with no postoperative complication (taurine group (T): 41% *vs.* placebo group (P): 45%, not significant (NS)). On per protocol analyses similar results were seen (T: 39% *vs.* P: 44%, NS).

At one year the number of overall medical comorbidities was similar between the groups. Functional ability, measured by Barthel ADL index, did not differ between the groups during the first year after surgery.

Overall mortality during the first year after surgery did not differ between the groups, as shown by the Kaplan Meier curve on intention-to-treat (Figure 2) or on per protocol analysis (Log-rank = 0.74, curve not shown). Overall mortality and mortality divided as in-hospital, at 3, at 6, at 9 and at 12 months follow up did not differ between the groups in the intention to treat analysis (Table 4). Per protocol analyses on mortality revealed similar results (data not shown).

#### 2.1.3. Postoperative Surgical Stress Response

Data on the surgical stress response are shown in Table 2.The inflammatory response to surgery, as determined by WBC, CRP and IL-6, was similar in the taurine and placebo group.

Among plasma antioxidant parameters (taurine, vitamin C, vitamin E, β-carotene and glutathione), only plasma taurine was different between the groups with higher levels in the taurine group. During the postoperative period, a slight increase in plasma β-carotene was seen in the placebo group, as shown by a trend in change over time with GEE analysis (Beta: −0.06; 95% CI: −0.11–0.00; *p* = 0.05).

In terms of oxidative stress, 8OHdG levels were significantly lower in the taurine group (Beta: −0.54; 95% CI: −1.08–−0.01; *p* = 0.04), the lactate/pyruvate ratio tended to be lower (Beta: −1.10; 95% CI: −2.33–0.12; *p* = 0.08) and the increase in MDA was less pronounced after surgery than in the placebo group. GEE analysis showed that the average difference of MDA over time (Beta: −0.45; 95% CI: −1.64–0.74; *p* = 0.46) did not differ between the groups, but the change over time in MDA did (Beta: 1.58; 95% CI: 0.00–3.15; *p* = 0.05). For the other oxidative stress parameters, oxLDL/apoB100 ratio and F2-Isoprostane, no difference between the groups was observed.

### 2.2. Discussion

This study is the first to show data on the effect of oral taurine supplementation on postoperative outcome in humans. Although peri-operative taurine supplementation attenuated postoperative oxidative stress, it did not improve morbidity and mortality in the elderly hip fracture patient. The absence of any effect of taurine on morbidity and mortality cannot be explained by insufficient enteral uptake or low biological availability, since taurine supplementation resulted in an almost nine-fold increase in its plasma level.

Our reported in-hospital mortality rate (T: 10% *vs.* P: 9%) is similar to that of Symeonidis *et al.* [14], but higher than the 5.4% in the study of Franzo *et al.* [1]. The latter could be explained by the older age of the patients included in the present study [15]. Our one year mortality rate is similar to that reported by Franzo *et al.* [1] and Gunasekera *et al.* [16]. The strong influence of patient case-mix variables, e.g., patient characteristics like gender or age, on mortality in hip fracture patients makes it difficult to influence mortality rates with a nutritional intervention [1,15,16].

A recent Cochrane review on nutritional intervention in elderly hip fracture patients by Avenell *et al.* showed no effects on mortality and little on morbidity. However, they concluded that most studies probably were underpowered to draw conclusions on outcome and did not use proper methodology [17]. Some more recent nutritional intervention studies in hip fracture patients showed promising effects on postoperative outcome, such as fewer complications and shorter length of hospital stay, but again most studies were powered only for evaluating changes in blood parameters [18,19,20]. A recent small study compared individualized nutritional supplementation in hip fracture patients to normal hospital diet, and showed that individualized supplementation lowered oxidative stress parameters, such as advanced oxidation protein products and MDA. Furthermore, these parameters were correlated with reduced length of hospital stay [19].

Taurine supplementation lowered postoperative oxidative stress in elderly hip fracture patients, as shown by reduced oxidative DNA damage (lower urinary 8OHdG), an attenuated response of lipid peroxidation (blunted plasma MDA response) and a trend towards a favorable balance of aerobic to anaerobic metabolism (slightly lower lactate/pyruvate ratio). This is in line with other demonstrations of the effectiveness of antioxidants in decreasing oxidative DNA damage in the frail elderly population. Comparing supplemental vitamin E *versus* placebo to counteract oxidative DNA damage, Chin *et al.* stratified their population between healthy young and older (over 50 years) individuals. In the older group, six months vitamin E supplementation decreased urinary 8OHdG compared to the placebo group, suggesting a reduction in oxidative DNA damage [21]. In a forearm ischemia/reperfusion model of oxidative stress in healthy aged individuals (61 to 75 years of age), tart cherry juice, containing flavonoids as antioxidants, lowered urinary 8OHdG levels to a level similar to that we found (1.8 *vs.* 2.0 μmol/mol creatinine in placebo group) [22]. The use of antioxidants apparently can reduce oxidative stress related DNA damage in the elderly, but whether this is of clinical relevance remains to be elucidated.

It is difficult to ascertain the oxidative status of our elderly patients on admission, as there is a lack of reliable reference values for oxidative parameters and of standardization of the different assays used. Preoperative 8OHdG levels in our patients are in line with other studies in healthy elderly people. For example, in healthy Koreans above the age of 60 years, similar urinary 8OHdG levels (mean 6.2 μg/g creatinine = 2.5 nmol/mmol creatinine) were observed using a similar assay [23]. For the lactate to pyruvate ratio a cut off value is given by Landow, who defines a ratio above 10:1 as discriminative for increased anaerobic metabolism due to oxygen deficits [24]. In our patients, the lactate to pyruvate ratio in the preoperative setting already was far above 10:1 suggesting increased anaerobic metabolism. Our results are thus suggestive of oxidative stress after a hip fracture, but if this was preexistent or the result of the hip fracture remains to be resolved.

The patients in the current study were surprisingly well nourished on admission, as assessed by the Minimal Nutritional Assessment, and had adequate nutritional intake during admission, as assessed using the “Nutrition Day in Europe form 3b”. A minority of the patients was at risk of malnutrition or was malnourished. The lack of any effect of taurine on postoperative outcome may have been obscured by the well-nourished state. Indeed, Koren-Hakim *et al.* showed that the lower the nutritional status on admission in elderly patients with a hip fracture, the higher the risk of postoperative mortality [25]. Furthermore, in recent studies the positive results of nutritional interventions in the elderly hip fracture population have been achieved in patients who were moderate to severely malnourished on admission [19,26].

A limitation of the current study is that the oxidative stress parameters were only measured in a subgroup of sixty patients and therefore are not representative for all patients. Although still a substantial number of patients, conclusions from these parameters related to outcome should be made with caution.

Finally, in the present study, taurine supplementation lowered oxidative stress, but it did not reduce postoperative morbidity and mortality in elderly hip fracture patients. Oxidative stress may not have been reduced enough to influence outcome and more research is warranted on the role of postoperative oxidative stress in postoperative outcome.

## 3. Materials and Methods

### 3.1. Patients

From March 2008 until July 2010, patients aged 75 years or older with a primary hip fracture and who were scheduled for surgery and admitted to the Medical Center Alkmaar (MCA, Alkmaar, The Netherlands) or to the Red Cross Hospital Beverwijk (RKZ, Beverwijk, The Netherlands) were included in the study. Exclusion criteria were inability to receive oral intake, major malabsorption, severe renal insufficiency (creatinine clearance < 30 mL/min), and participation in another trial. The study was approved by the local regional Medical Ethics Committee and conducted according to the Declaration of Helsinki (as revised in 1983). Written informed consent was obtained from all patients. The study is registered at www.clinicaltrials.gov (NCT00497978).

### 3.2. Randomization, Blinding and Nutritional Intervention

The patients were randomly allocated to one of the two groups receiving either oral taurine or placebo (microcrystalline cellulose) capsules. The intervention started before surgery within 24 h after hospital admission and continued up to six days after surgery. A computerized randomization table using block randomization of 30 patients per block, generated by a local statistician, was used by the pharmacological department to label the capsules for our intervention. From the pharmacological department, the investigators received bottles with capsules in consecutive numbers, containing taurine or placebo capsules, with a bottle for each patient. Investigators, patients, medical and nursing staff were unaware of intervention allocation, since the taurine and placebo capsules were identical in appearance, color and smell. The randomization code was broken after completion of the study.

Oral administration of taurine up to 6 g/day to healthy subjects or patients is well tolerated [27,28]. In the present study, taurine or placebo capsules were administered three times a day (scheme: 2-1-2 capsules of 1.2 gram taurine or placebo) to reach a 6 g daily dosage and was started before surgery. When patients were released from the hospital before the end of the supplementation period, the study protocol was continued at (a nursing) home by or under supervision of the principal investigator (Alexander P.J. Houdijk).

The first contact with eligible patients was at the emergency ward or at the acute admission ward of the hospital. After receiving informed consent, the patients received the first two capsules of the nutritional intervention and were interviewed for a Mini Nutritional Assessment (MNA) and the Barthel Activities of Daily Living Index (Barthel ADL index) questionnaire. In the postoperative period dietary intakes as breakfast, lunch and dinner were scored using the “Nutrition Day in Europe form 3b”.

### 3.3. Study Outcomes

The primary outcome of the study was the effect of oral taurine supplementation on postoperative morbidity and mortality during hospitalization and the first year after surgery. All postoperative complications were recorded. After discharge, patients’ follow up was at three months intervals until one year postoperatively. At follow up morbidity and mortality was registered. Two investigators, who were unaware of treatment allocation, independently determined the occurrence of postoperative complications and morbidity.

Secondary outcomes focused on possible mechanisms of action of taurine, e.g., reducing postoperative oxidative stress or reducing the postoperative inflammatory response. Therefore blood samples were drawn on admission, and on the 1st and 5th postoperative days.

### 3.4. Laboratory Measurements

Laboratory assessments were done in a predefined group of 60 patients using numbers two and three of the randomization blocks. Blood samples were drawn on the emergency ward at admission before receiving the study capsules, and between 08.00 and 10.00 a.m. on the first and fifth postoperative day.

#### 3.4.1. Inflammatory Parameters

*WBC* and *Hb* were measured using a Sysmex XE2100 analyzer (Sysmex Corporation, Kobe, Japan). *CRP* was measured on a Synchron analyzer (Beckman Coulter, Fullerton, CA, USA). *IL-6* was measured with an ELISA technique (Pelikine human IL-6, Sanquin, Amsterdam, The Netherlands; detection limit: 0.5 pg/mL). F2-Isoprostanes in urine, as described in the antioxidant/oxidant parameters section, were also measured as an inflammatory parameter.

#### 3.4.2. Antioxidant/Oxidant Parameters

*Vitamin C* was determined spectrophotometrically. Activated carbon oxidized vitamin C to dehydroascorbic acid, followed by coupling to 2.4-dinitrophenylhydrazin in 85% H_2_SO_4_. A formed stabile solution of orange color has a maximum absorption of 545 nm. Vitamin E and beta-carotene were determined as described by Miller and Yang [29]. Total blood glutathione (GSH) was measured by reverse-phase HPLC on a Dionex system (Dionex, Voisins Le Bretonneux, France) with electrochemical detection. Oxidized LDL (ox LDL) and apolipoprotein B100 (apoB100) were determined as described by van der Zwan *et al.* [30] and the oxLDL/apoB100 ratio was calculated. Plasma malondialdehyde (MDA) concentrations were determined by HPLC with fluorescence detection as described by Van De Kerkhof *et al*. [31]. The intra- and inter-assay coefficients of variations (CV) were 3.5% and 8.7%, respectively. F2-Isoprostanes in urine were determined by LC-MS/MS as described by Fischer *et al.* [32]. The intra- and inter-assay CV were 4.8% and 10.1%. Urine levels of 8-hydroxy-2-deoxyguanosine (8OHdG) were determined as described by Fischer *et al.* [32]. Lactate and pyruvate concentration were analyzed using a conventional enzymatic method (Cobas-Mira Plus analyzer, Roche, Branchburg, NJ, USA). The lactate/pyruvate ratio was calculated. Plasma taurine was determined by reversed-phase HPLC as previously described [33].

### 3.5. Statistical Analysis

On the basis of the assumption of a 50% reduction in mortality rate at one year follow up, a 2-tailed α = 0.05 and a β = 0.20, a sample size of 2 times 118 patients was calculated.

Baseline characteristics and part of the outcome parameters, normally distributed continuous and ordinal variables, were analyzed with Student’s *t* tests for between-group comparisons. A part of the outcome parameters, nonparametric continuous and ordinal variables, were analyzed with Mann-Whitney *U* tests. Dichotomous variables of the outcome parameters were analyzed with χ^2^ or Fisher’s Exact tests. Group survival, from the admission date until date of death or date at loss in follow-up, was generated by the method of Kaplan Meier and compared by means of the log-rank test. The SPSS software (version 20.0, SPSS Inc., Chicago, IL, USA) was used for statistical analysis.

Generalized estimating equations (GEE) were used to compare changes in postoperative inflammatory parameters, Barthel ADL-indices, Nutrition Day in Europe scores and taurine plasma levels over time between the taurine and placebo group [34]. GEE is a linear regression technique, suitable for analyzing data from a longitudinal study in which outcome variables are repeatedly measured in each individual. The difference between the taurine and placebo group over time were analyzed in two ways. In the first analysis the average difference over time between the groups was analyzed, while in the second analysis it was investigated whether the difference between the groups changed over time. This was investigated by adding an interaction term between time and group to the GEE model. All GEE analyses were adjusted for baseline differences between the groups in the particular outcome variable. Furthermore, the effect of patient and treatment related factors (age, male/female, BMI, nutritional status and type of nutritional intervention) was determined by GEE analysis. GEE analyses were performed with STATA^®^ (version 11.0).

For all statistical analyses a *p*-value <0.05 was considered statistically significant. Intention to treat and per protocol analyses were performed. For per protocol analysis a minimum intake of 31.2 grams of taurine or placebo (1 capsule preoperatively + 5 capsules times 5 days postoperatively = 26 × 1.2 = 31.2 grams) was defined as a cut-off point for protocol adherence.

## 4. Conclusions

Taurine supplementation in elderly hip fracture patients lowered postoperative oxidative stress. This was shown by a reduction in oxidative DNA damage, an attenuated response on lipid peroxidation and a trend towards a favorable balance of aerobic to anaerobic metabolism.

Our elderly hip fracture patients were surprisingly well nourished on admission with adequate nutritional intake as well. Perhaps due to their well-nourished state, taurine supplementation did not have an effect on postoperative morbidity and mortality in these elderly hip fracture patients.

## Figures and Tables

**Figure A1:**
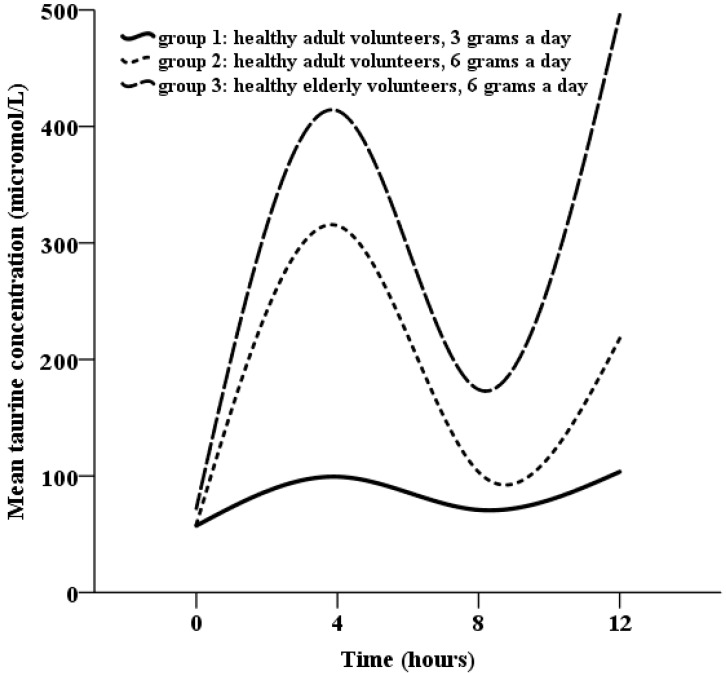
Results of a dose finding on taurine dosage.

## Appendix

A dose finding was performed before the start of the clinical trial to decide on the dosage to give. Based on a literature search, as described in the method section of the manuscript, it was known that dosages up to six grams a day could be safely administered. We did not find in the literature evidence for the rise and decline of plasma taurine levels in a regimen suitable for our clinical trial. Therefore, we performed a dose finding to decide on the dosage and regimen to give (Figure A1).

First, we administered two types of dosages to healthy adult volunteers. Three healthy adult volunteers received 1 gram of taurine every eight hours (regimen of 3 grams a day) and another three healthy adult volunteers received 2 grams of taurine every eight hours (regimen of 6 grams a day). The aim was to have at least a 100% rise in pasma taurine levels and a decline that would not be in the normal plasma taurine level range. Blood samples for plasma taurine levels were drawn at an interval of four hours, with the first blood sample drawn just before the intake of the first intake of taurine. Based on the literature and on the first part of our dose finding we preferred to give a regimen of 6 grams a day. To complete the dose finding we administered the regimen of 6 grams a day to healthy elderly volunteers. Based on the total dose finding we decided to administer 6 grams a day in the clinical trial, where we found sustained elevated plasma taurine levels.
